# *De novo* vesicle formation and growth: an integrative approach to artificial cells

**DOI:** 10.1039/c7sc02339a

**Published:** 2017-10-26

**Authors:** Ahanjit Bhattacharya, Roberto J. Brea, Neal K. Devaraj

**Affiliations:** a Department of Chemistry and Biochemistry , University of California, San Diego , La Jolla , CA 92093 , USA . Email: ndevaraj@ucsd.edu

## Abstract

The assembly of artificial cells provides a novel strategy to reconstruct life's functions and shed light on how life emerged on Earth and possibly elsewhere. A major challenge to the development of artificial cells is the establishment of simple methodologies to mimic native membrane generation. An ambitious strategy is the bottom-up approach, which aims to systematically control the assembly of highly ordered membrane architectures with defined functionality. This perspective will cover recent advances and the current state-of-the-art of minimal lipid architectures that can faithfully reconstruct the structure and function of living cells. Specifically, we will overview work related to the *de novo* formation and growth of biomimetic membranes. These studies give us a deeper understanding of the nature of living systems and bring new insights into the origin of cellular life.

## Abstract 2

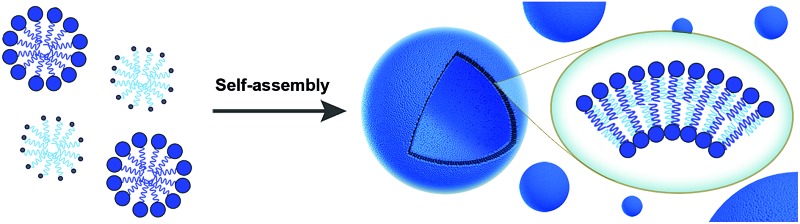
The assembly of synthetic membranes provides a powerful tool to reconstruct the structure and function of living cells.

## 1. Introduction

Self-assembly is central to the very organization of life.^1^ It is believed that self-assembly played a key role in the origin of life by organizing building blocks for more complex structures. Non-covalent interactions like hydrogen bonding, van der Waals forces, and dipolar interactions play major role in this process.^2^ A wide variety of building blocks ranging from simple amphiphiles, polymers, crystals, and nanostructures are capable of self-assembly.^3^ Every living cell is bounded by a highly dynamic and semi-permeable cell membrane, which is mainly composed of amphiphilic phospholipids. In aqueous solutions, phospholipids spontaneously self-assemble into membrane-bound structures called vesicles. This process is largely driven by hydrophobic interactions between the non-polar fatty acyl chains of the phospholipid molecules, which segregates them from the aqueous bulk solution. Phospholipids are quite complex amphiphiles and may not have evolved without a sophisticated biochemical machinery.^4^ Previous studies have suggested that the earliest membranes were composed of simple single-chain amphiphiles like fatty acids and fatty alcohols derived from geochemical processes and extraterrestrial sources (meteorites and comets).^5^ Such vesicles have been shown to be capable of harboring prebiotically plausible molecules and sustaining a few simple biochemical reactions.^6^ However, an increase in the complexity of membrane-forming molecules must have been necessary to the early evolution of life. It has been hypothesized that the inclusion of phospholipids provided a competitive edge to protocells containing them.^7^ Therefore, it is important to understand the plausible pathways which led to earliest emergence of phospholipids and other complex amphiphiles.

Phospholipid membranes have been extensively utilized for studying biological membranes, as drug delivery vehicles, monitoring drug–receptor interactions, and as components of microreactors.^8^–^10^ Physicochemical studies on the properties of phospholipid membranes also comprise a rich literature.^11^ More recently, there has been a significant interest in using phospholipid vesicular structures as model systems for bottom-up construction of synthetic life.^12^,^13^ We envision that this goal can be successfully accomplished through the use of an integrative approach based on increasing complexity in the following hierarchical manner: (1) exploration of facile chemoselective reactions for generation of phospholipids and other membrane forming molecules, (2) growth and division of vesicles driven by a catalytic process, (3) attributing functionalities to the artificial cell membranes to mimic biological processes, (4) use of vesicles as microreactors for gene expression and simple biochemical reactions, (5) integrating growth, division and metabolic events to genetic circuits, and (6) demonstration of Darwinian evolution. In this perspective, we will discuss the accomplishments in the area of vesicle-based synthetic cells, with a special emphasis on *de novo* construction of biomimetic membranes.

## 2. Self-assembly driven by simple chemical reactions

### 2.1. *De novo* membrane formation: inspiration from biology

**Fig. 1:**
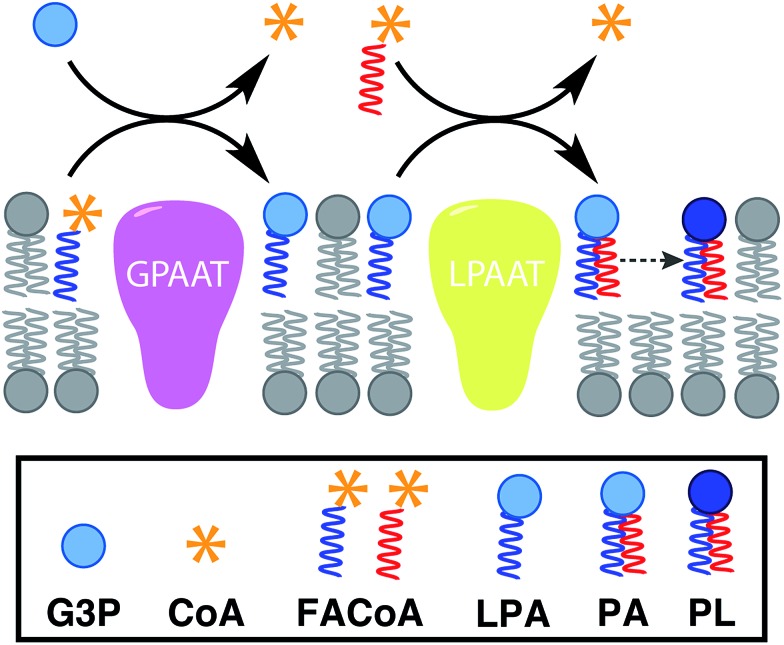
*De novo* formation of phospholipids based on the biosynthetic pathway, which involves multiple membrane-bound enzyme-catalyzed steps, substrates and cofactors [GPAAT: glycerol-3-phosphate: acyl-CoA acyltransferase, LPAAT: lysophosphatidic acid acyltransferase, G3P: glycerol 3-phosphate, CoA: coenzyme A, FACoA: fatty acyl coenzyme A, LPA: lysophosphatidic acid, PA: phosphatidic acid, PL: phospholipid].

In nature, membrane-forming lipids are synthesized in multiple enzyme-catalyzed steps and involves several cofactors (Fig. 1).^14^ Over the last years, there has been considerable effort in reconstituting these biochemical reactions in liposomes, albeit with varying degrees of success.^15^ The key enzymes for lipid synthesis pathways are membrane-bound proteins,^14^ and hence difficult to reconstitute in functional form. The reliance on integral membrane proteins implies that new biological membranes cannot be synthesized without pre-existing membranes. For the bottom-up design of an artificial cell, a fundamentally different strategy is desirable. To eliminate the necessity for pre-existing membranes, the reactive precursors should themselves be non-membrane forming and can react among themselves in a chemoselective fashion to form membrane-forming materials. We refer to this strategy as *de novo* membrane formation. Inspiration for this strategy was drawn from the Land's Cycle – a process which takes place in biological membranes for phospholipid remodeling.^16^ In this cycle, non-membrane forming single-chain amphiphiles–lysophospholipids and fatty acyl-CoA thioesters – are coupled together by the enzyme lysophospholipid acyltransferase (LPLAT). In early seminal work, Deamer and co-workers showed that membranes can be reconstituted from water-soluble precursors by utilizing the LPLAT activity of solubilized liver microsomal membranes.^17^,^18^ However, the system was not well-defined and not suitable for using as such for bottom-up membrane assembly. It will be more desirable to utilize simple, robust and high-yielding reactions for this purpose. In the following sections, we will discuss the applications of bioorthogonal and chemoselective coupling reactions for *de novo* membrane assembly.

### 2.2. Click chemistry-based approaches

**Fig. 2:**
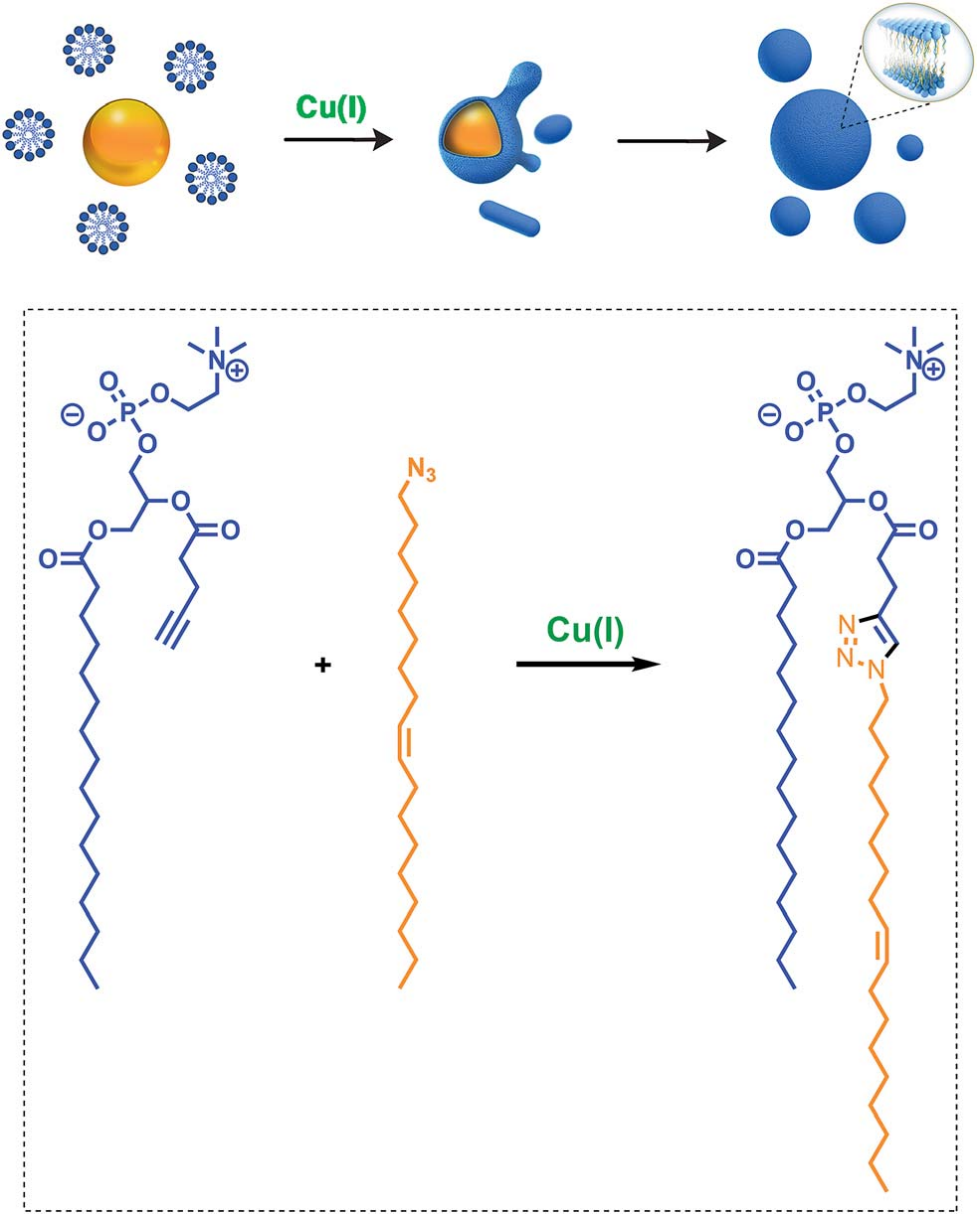
Biomimetic formation of phospholipid membranes driven by a copper(i) catalyzed azide–alkyne cycloaddition (CuAAC) reaction. Reactive alkyl azide oil droplets (orange) interact with alkyne lysophospholipid micelles (blue) in aqueous solution to form an emulsion. Addition of the copper(i) catalyst (green) triggers the cycloaddition reaction, which takes place primarily at the interface between the oil droplets and the aqueous phase. Over time, the droplets are consumed and replaced with vesicular phospholipid membrane structures, both spherical and tubular. Bottom: reaction scheme of the CuAAC, which is the key process for the formation of phospholipid membranes.

Given the relevance of mimicking biological systems, several groups have recently explored methodologies for the *de novo* formation of synthetic lipid membranes from reactive precursors. The first example based on a bioorthogonal approach^19^ utilized the copper(i) catalyzed azide–alkyne cycloaddition (CuAAC)^20^–^22^ reaction to drive the assembly of membranes (Fig. 2).^19^ This system employs a biomimetic coupling reaction to join an alkyne-functionalized lysophospholipid and an alkyl azide in presence of a copper catalyst, which generates an abiological triazole-linked phospholipid. As expected, neither the alkyne nor azide precursors formed membranes in aqueous solution. However, the triazole-containing phospholipid product, when hydrated, readily formed membrane vesicles. *In situ* membrane formation occurs spontaneously and does not require pre-existing membranes to house catalysts or precursors. Bioorthogonal click chemistry reactions take place at the interface of insoluble alkyl azide emulsion droplets and the monolayers of alkyne lysolipid that presumably coat the droplets. Steady-state fluorescence anisotropy measurements showed that the triazole phospholipid membranes closely resemble their natural counterparts in fluidity properties. Overall, the strategy is biomimetic in that it proceeds in aqueous medium with high specificity and a lack of background reactions in the absence of a catalyst.

Follow-up work by Hardy *et al.* demonstrated that the CuAAC reaction can be light-triggered by ruthenium tris-bipyridine [Ru(bpy)_3_^2+^] complex under mild aqueous conditions.^23^ The ruthenium-copper electron transport chain was found to drive the *in situ* formation of phospholipids in a high yielding manner, which subsequently self-assemble into synthetic membrane vesicles. Konetski *et al.* further demonstrated that the incorporation of a photoinitiator complex into the CuAAC reaction can be used to achieve spatiotemporal control over vesicle assembly.^24^ It was also shown that the vesicle size and morphology could be controlled by the intensity of light. We foresee future applications of this simple photoinduced vesicle formation technology in the controlled construction of complex synthetic advanced cells.

In a separate work, Zhou *et al.* showed that a photo-initiated thiol-yne click chemistry reaction could be used to rapidly synthesize dithioether analogs of phospholipids in aqueous solution.^25^ The resulting lipids were shown to possess physical properties comparable to natural phospholipids. Their resistance to phospholipases could potentially be useful for drug delivery applications.

### 2.3. Chemoselective ligation methodologies

**Fig. 3:**
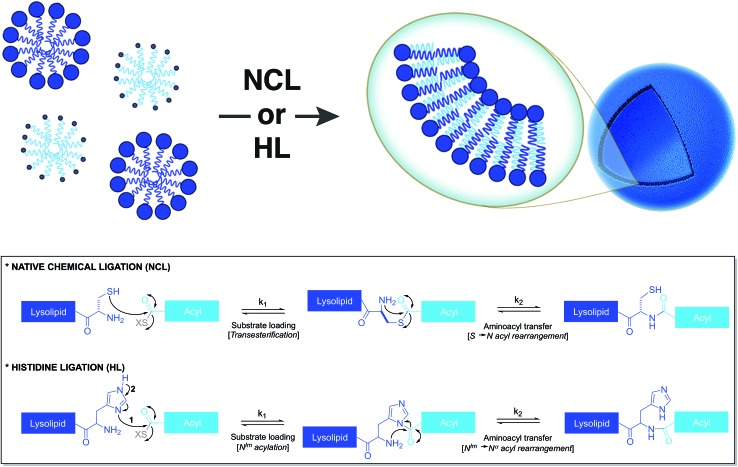
Nonenzymatic and chemoselective approaches to spontaneously generate phospholipid membranes from water-soluble precursors. Bottom: mechanisms for the native chemical ligation (NCL) and histidine ligation (HL) reactions.

Based on a similar strategy, Brea *et al.* utilized native chemical ligation (NCL)^26^,^27^ to drive the chemoselective coupling between a cysteine-functionalized lysophospholipid and a water-soluble fatty acyl thioester (Fig. 3).^28^ This approach overcame some of the drawbacks of the CuAAC-based approach. For example, it did not require the use of any catalyst and both precursors were water-soluble. Unlike the NCL reaction between peptide counterparts, the reaction between the amphiphilic precursors was found to be very rapid and high yielding. It went to completion within 30 min with full conversion. This rate enhancement and high efficiency can be attributed to the hydrophobic interaction between the reactive partners, which brings them in close proximity. The generated amidophospholipids were shown to have physical properties like their natural counterparts. The reaction was also shown to be compatible with biomolecules and biologically relevant conditions.

Brea *et al.* further showed that histidine ligation (HL)^29^,^30^ can be used in an analogous manner to *de novo* generate amide-linked phospholipids (Fig. 3).^31^ In particular, the catalytic role of the imidazole ring of a histidine-functionalized lysolipid was utilized to drive its coupling to a fatty acyl thioester, which affords a new class of amidophospholipids. The corresponding phospholipids can spontaneously self-assemble into micron-sized vesicles. Moreover, the selectivity, the high reaction rate and the biocompatibility of this methodology are key features that make it a powerful tool for the efficient encapsulation of biomacromolecules, such as proteins. Remarkably, the HL reaction, which was previously found to be quite inefficient for peptide ligation, proceeded with high efficiency in an amphiphilic milieu.

In principle, any simple, robust, and chemoselective coupling reaction that takes place under mild aqueous condition could be used to generate phospholipids *in situ*. This opens up the opportunity to build a diverse toolbox for generating lipid membranes chemically.

### 2.4. Reversible imine condensation

**Fig. 4:**
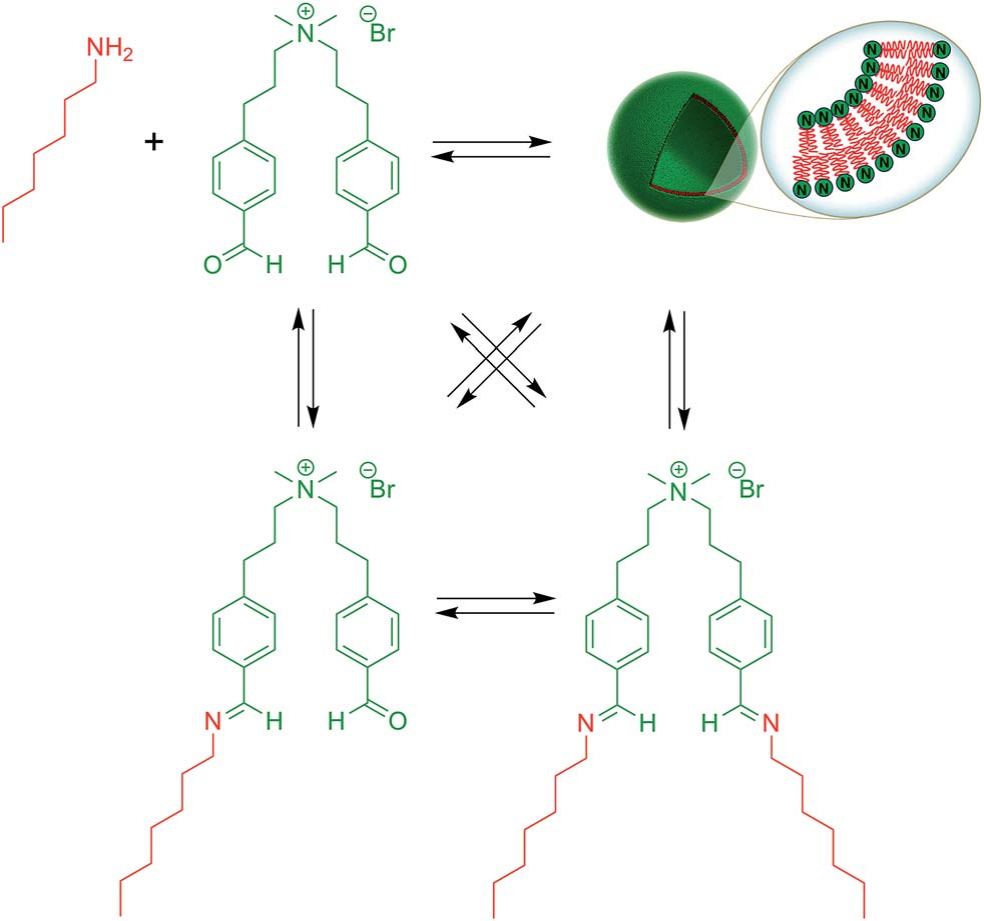
Dynamic vesicle formation based on covalent imine bonds.

Over the last years, dynamic covalent chemistry (DCvC) has been used to construct complex supramolecular assemblies from discrete molecular building blocks using chemical reactions carried out reversibly under conditions of equilibrium control.^32^ An attractive strategy to create a dynamic vesicular system is to exploit the reversible nature of imine condensation.^33^ In this particular case, an aldehyde and an amine can be condensed to an imine at alkaline pH and the latter can be hydrolyzed at acidic pH.^33^ The group of van Esch employed this simple and elegant idea to demonstrate the *de novo* emergence of vesicular structures, whose formation and disassembly can be reversibly regulated simply by changing the pH (Fig. 4).^34^ The introduction of dynamic covalent imine bonds represents a new strategy for generating dynamic vesicular architectures, which are highly interesting for controlled drug delivery because of their easy formation, fast dissociation, and switchable morphology. Using an analogous reversible imine–aldehyde reaction, Takakura *et al.* were able to demonstrate the transformation of submicroscopic micelles into GVs.^35^ In this particular example, it was shown that the hydrophobic environment of micelles led to the release of a long-chain amine from an imine. Subsequently, the long-chain amine reversibly forms a new imine with a single-chain aldehyde to generate a double-chained membrane-forming amphiphile.

More recently, the Yi group used a similar approach to develop a novel class of pH-responsive vesicular structures from an equimolar mixture containing octyl amine and the supra-amphiphiles 1-[10-(4-formyl-phenoxy) decyl] pyridinium (FDP) or 1-methyl-3-[10-(4-formyl-phenoxy) decyl] imidazolium (FDI).^36^ The efficient encapsulation of Nile Red and its rapid release in a low pH environment demonstrated the capabilities of these unilamellar vesicles to act as potential drug delivery systems in a biological environment.

## 3. Vesicle growth and division promoted by catalytic membranes

Growth of vesicle membranes composed of single-chain amphiphiles like fatty acids has been demonstrated simply by addition of fresh monomers.^37^ However, owing to the less dynamic nature of phospholipid membranes, *in situ* generation of membrane-forming material is required for their growth.^12^ This can be accomplished either by a membrane-embedded catalyst or by a soluble catalyst encapsulated within the vesicle lumen. Also, there are examples where the hydrophobic interior of the membrane itself has been shown to play a catalytic role to drive a reaction. Generation of excess membrane leads to an imbalance in the surface area to volume ratio, which subsequently leads to budding and fission events. This is reminiscent of the division events which take place in L-form bacteria.^38^

**Fig. 5:**
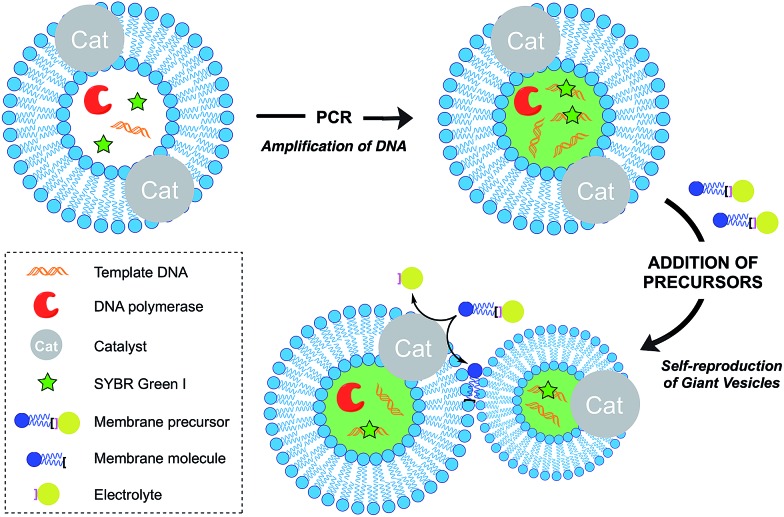
Schematic representation of the amplification of DNA within a self-reproducible giant vesicle (GV). Addition of membrane precursors causes the growth and spontaneous division of the GVs, as well as the efficient distribution of DNA to the daughter GVs.

Over the last decade, the Sugawara group has investigated this problem thoroughly. For instance, Takakura *et al.* reported the self-reproduction of giant vesicles (GV) driven by an imine hydrolysis reaction taking place in a membrane environment.^39^ Kurihara *et al.* also showed that the growth and division of giant vesicles composed of various phospholipids and a cationic amphiphile can be coupled with the polymerase chain reaction (PCR) taking place inside the GVs (Fig. 5).^40^ In the first step, PCR led to the amplification of DNA encapsulated in the GVs. The amplified DNA interacted with a cationic membrane amphiphile to form a “pseudo-catalyst” complex which served to facilitate the hydrolysis of a bolaamphiphilic membrane precursor. Consequently, new membrane material was generated, thus leading to membrane growth and division. In subsequent work, Kurihara *et al.* also showed that a model cell cycle can be developed using a similar vesicular system.^41^ However, in all the previous examples, membrane growth led to dilution of the catalyst and membrane formation could not be sustained for more than a few rounds. Ideally, the reproduction of the membrane should be coupled with the reproduction of the catalytic species. This can be accomplished by an autocatalytic mechanism.

**Fig. 6:**
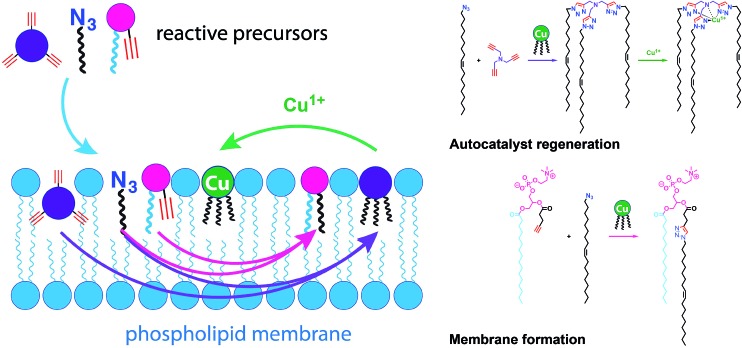
Self-reproducing system that can drive the repeated synthesis and growth of phospholipid membranes by using a CuAAC reaction. The regeneration of membrane-bound autocatalysts continually induces the formation of triazole phospholipids, mimicking natural membrane generation.

Simple autocatalytic reactions have been utilized in the past to drive the growth of membrane-bound vesicles. Wick *et al.* demonstrated the growth of oleic acid vesicles driven by the autocatalytic hydrolysis of oleic anhydride droplets.^42^ Seeking a catalytic strategy to obtain vesicle self-reproduction, Hardy *et al.* recently reported the use of a self-replicating catalyst,^43^ which can continuously drive phospholipid formation by CuAAC reaction over many generations in a high yielding manner (Fig. 6).^43^ The catalytic species is a membrane-embedded tris-(lauryl triazole) amine (TLTA)-Cu(i) complex, which uses lauryl (dodecyl) azide for its own generation, as well as for the generation of triazole-containing phospholipids. Phospholipid formation led to growth of the membrane and occasional division events. In principle, such a system should be able to sustain efficient membrane reproduction if the precursors are continuously replenished.

The current methods of artificial vesicle growth and division lack the sophistication or spatiotemporal control found in living cells.^37^ However, we envision that these limitations can potentially be overcome by coupling various strategies of *de novo* membrane formation with finely tuned physicochemical triggers or genetic circuits. Additionally, it would be desirable to employ non-amphiphilic water-soluble precursors to rule out non-specific interactions arising from incorporation of amphiphiles into the membranes.

## 4. Remodeling artificial membranes

**Fig. 7:**
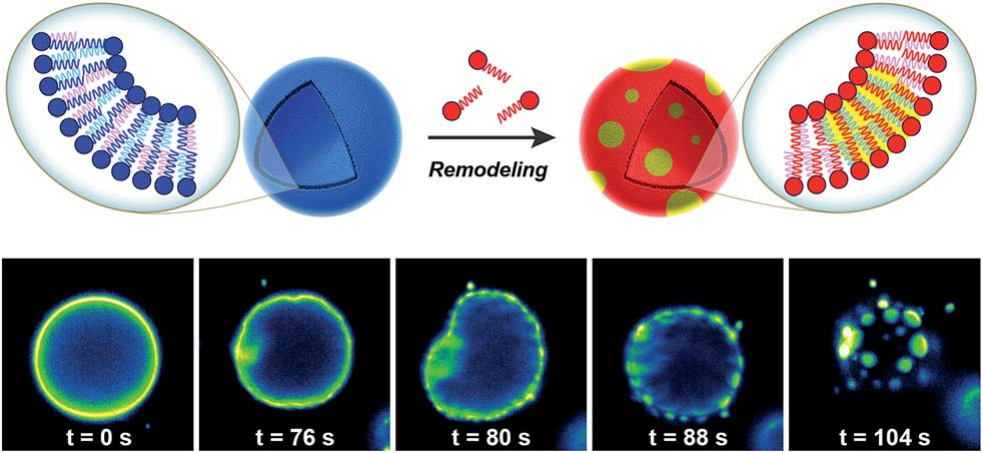
Lipid microdomain formation induced by *in situ* nonenzymatic remodeling of phospholipid membrane architecture. GUVs composed of unsaturated phospholipids are remodeled by lipid tail exchange reactions with a saturated cysteine-modified lysophospholipid, leading to the formation of a new class of GUVs composed of a mixture of unsaturated and saturated lipids. After addition of the corresponding lysophospholipid, membrane remodeling rapidly takes place and subsequent microdomain formation is observed, as indicated by the partitioning of Texas Red® DHPE dye into discrete circular regions of the membrane.

Biological membranes are highly dynamic structures. Membranes can be remodeled in response to various stimuli by altering the composition of the phospholipids. This results from the transacylation activities of membrane-bound enzymes in the presence of different lysolipid, phospholipid, and fatty acid precursors.^16^ Several permutations of phospholipids can be generated by this remodeling, which allows cells to modify membrane properties. Therefore, the importance of this process underscores the need for robust strategies to remodel lipid composition in artificial membranes. Researchers have recently developed simplified membranes that mimic how native phospholipid membranes are remodeled. For instance, Brea *et al.* have shown that reversible chemoselective reactions can be harnessed to achieve nonenzymatic spontaneous exchange and remodeling of both lipid tails and head groups in synthetic membranes.^44^ Using this approach, they demonstrated the ability of phospholipid remodeling to trigger changes in vesicle spatial organization, composition, and morphology, as well as the recruitment of proteins that can affect membrane curvature. The feasibility of remodeling phospholipid membranes was initially demonstrated by using reversible transthioesterification/acyl shift reactions for the construction of dynamic membranes that modulate their physical and chemical properties through efficient exchange of their phospholipid tails. Remarkably, *in situ* membrane remodeling by lipid tail exchange also lead to subsequent micrometer scale changes in membrane morphology and the formation of distinct lipid microdomains (Fig. 7).

In addition to controlling membrane fluidity, Brea *et al.* were able to modulate the membrane association of amphiphysin^45^,^46^ and epsin 1 47 by using a lysophospholipid possessing a negatively charged head group.^44^ Amphiphysin and epsin 1 are proteins that bind to negative phospholipid membranes and induce positive curvature of the surface.^45^–^47^ After *in situ* remodeling of membranes, the corresponding protein was added, and dramatic curvature events in the negatively charged vesicles were observed.

We foresee future applications of the *in situ* lipid remodeling approach for studying the specific effects of lipid fragment exchange in minimal model membranes. An interesting application would be devising an artificial approach to the reconstitution of whole lipid rafts.

## 5. Reconstruction of lipid-synthesizing machinery in vesicles

Linking gene expression with phospholipid membrane formation is one of the highly sought after goals of bottom-up synthetic biology.^48^ The ability to form phospholipids by an enzyme-catalyzed reaction provides control over membrane assembly on a genetic level. Over the last few decades, there has been significant interest and effort for developing robust strategies to drive lipid membrane synthesis from well-defined enzymatic systems. Most of the work to date has been focused on reconstitution of the *de novo* (Kennedy) pathway of phospholipid synthesis.^49^ In this pathway, phospholipid synthesis takes place by adding various substituents to glycerol-3-phosphate (G3P) in several enzyme-catalyzed steps.

**Fig. 8:**
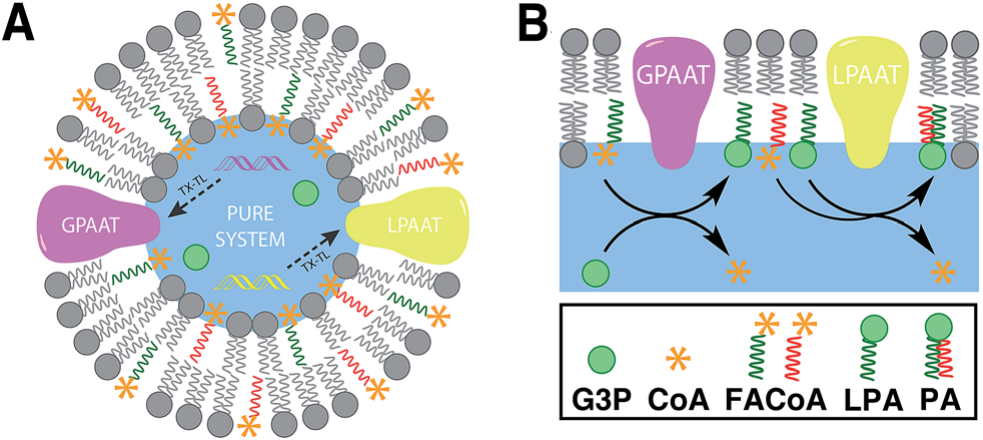
Construction of membrane proteins in minimal synthetic cells. (A) Schematic representation of a lipid-synthesizing liposome. The key membrane proteins (GPAAT and LPAAT) are expressed by using an encapsulated cell-free system (PURE system) in the presence of appropriate reactive precursors [TX–TL: transcription/translation]. (B) G3P is initially converted into LPA by using the protein GPAAT. Then, the resulting LPA is used as a substrate to catalyze the formation of PA using the protein LPAAT.

The Luisi group has produced extensive work in this area. In the first example, Schmidli *et al.* showed that phosphatidylcholine synthesis can be effected by using four purified membrane proteins bound to a liposome membrane.^50^ In a later work, Kuruma *et al.* demonstrated that vesicles encapsulating a cell-free transcription/translation (TX-TL) system (PURE® system) can express two membrane proteins (GPAAT and LPAAT), that can catalyze the formation of new phosphatidic acid (Fig. 8).^51^

Very recently, the Danelon group demonstrated that a total of eight *E. coli* enzymes for various acyl transfer and head group modification reactions can be expressed in the PURE® system and reconstituted in liposomes.^52^ A different approach was taken by Murtas, where he reported vesicle growth arising from internal fatty acid synthesis catalyzed by a single soluble bacterial enzyme complex FAS-B (type I).^53^

However, these approaches were met with low overall yields (<10%), thus limiting their scope of application towards vesicle growth and division.^48^ Also, *de novo* membrane formation was not demonstrated as the biosynthetic enzymes require pre-existing membranes to be functional. A major challenge to the encapsulation of the natural lipid synthesis machinery is the difficulty of reconstituting the various membrane-bound proteins involved in phospholipid biosynthesis. Continued supply of precursors and cofactors can be also challenging if they are charged and membrane impermeable. This difficulty can be partly alleviated by introduction of pore forming proteins to the membranes. We also believe that approaches radically different from the natural pathways can also be useful to approach to this problem. Linking enzymatic reactions with the coupling strategies discussed in the previous sections could help minimize the number of components used for lipid synthesis and possibly improve the efficiency.

## 6. Functionalization of artificial membranes

For the bottom-up construction of an artificial cell, a major goal will be the functionalization of the membranes with suitable proteins. In this section, we will discuss various methods of specific localization of both soluble proteins and integral membrane proteins to artificial membranes.

### 6.1. Mimicking membrane-anchored proteins

**Fig. 9:**
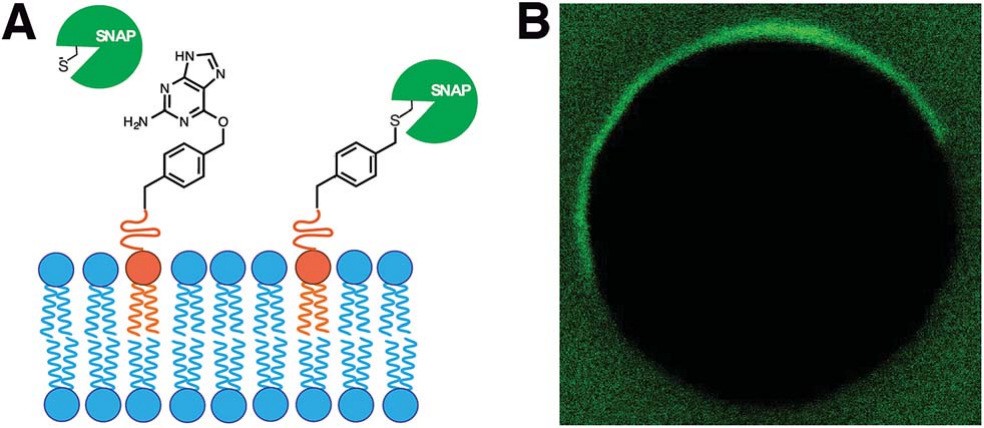
Membrane localization of proteins by SNAP-tag reaction with lipid-anchored substrates. (A) Schematic representation of the reaction between a SNAP-tag protein and a benzylguanine (BG)-functionalized phospholipid membrane. (B) Fluorescence microscope image showing spatiotemporally controlled membrane localization of an Alexa Fluor® 488 labeled SNAP-tag protein on a GUV.

In living cells, recruitment of soluble proteins to membranes is an important event in cellular signaling, cell recognition, and for affecting membrane curvature.^54^ This is accomplished by attaching the protein to a site-specific lipid anchor (*e.g.* palmitoylation, prenylation)^55^ or to a phospholipid head group [*e.g.* glycerophosphoinositol (GPI) anchor].^56^ Therefore, it would be interesting in synthetic biology to develop analogous tools for site-specific localization of proteins to artificial membranes. Rudd *et al.* described a methodology of covalent protein localization to giant unilamellar vesicle (GUV) membranes based on the chemoselective reaction between benzylguanine (BG)-functionalized phospholipid anchors and a SNAP-tagged GFP (Fig. 9A).^57^ It was also shown that the process could be spatiotemporally controlled using light with high precision (Fig. 9B).

Over the last years, van Hest group has been developing efficient strategies for controlling the recruitment of proteins to membranes. For instance, Peters *et al.* have recently exploited the non-covalent interaction between a Ni-NTA modified lipid and an oligo-His tagged protein for the latter's localization to GUV membranes.^58^ Exploiting the pH-dependent nature of this interaction, it was also shown that the binding could be reversibly switched by coupling the process with the enzymatic activity of alcohol dehydrogenase.

### 6.2. Reconstitution of transmembrane proteins

Reconstitution of membrane proteins is particularly interesting because of their diverse roles in signal transduction, transport, ligand binding, membrane architecture, and cellular adhesion.^54^,^59^,^60^ However, it is a challenging task to reconstitute integral membrane proteins in their functional form because of the poor aqueous solubility of their transmembrane domains.^61^ Conventional methods involve detergent solubilization, organic extraction or mechanical fragmentation followed by transfer to a liposome.^62^ Although several membrane proteins have been reconstituted using this technology, the process is very time consuming and residual detergents adhering to the liposomes can influence the protein's properties as well as subsequent analyses.^63^

**Fig. 10:**
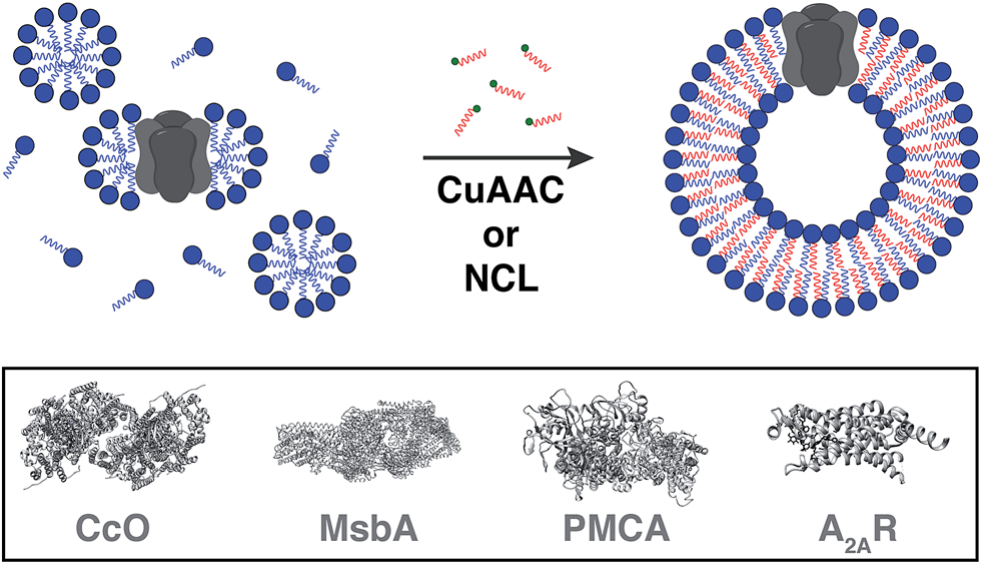
Model for spontaneous reconstitution of transmembrane proteins during *in situ* phospholipid membrane formation. The desired protein is initially solubilized with a synthetic mimic of a detergent to form micelle-solubilized protein complexes. Addition of the complementary reactive precursor and subsequent coupling results in the spontaneous generation of the corresponding proteoliposomes [CcO: cytochrome *c* oxidase, PMCA: plasma membrane calcium ATPase, A_2A_R: adenosine A_2A_R receptor].

Cole and co-workers tackled this problem by coupling bioorthogonal methods of *in situ* phospholipid membrane formation to spontaneous membrane protein reconstitution (Fig. 10).^64^ In this strategy, a functionalized (*e.g.* terminal alkyne or cysteine) lysophospholipid was imparted a dual role. In the first step, the lysolipid functioned as a detergent in solubilizing the protein in micelles. In the next step, the lysolipid reacted with a suitable partner (*e.g.* alkyl azide or fatty acyl thioester) in a bioorthogonal manner to generate a phospholipid which assembled to form membranes along with spontaneous reconstitution of the protein. CuAAC reaction was utilized to reconstitute commercially available cytochrome *c* oxidase (CcO)^65^,^66^ – a transmembrane protein universally important in aerobic respiration.^67^ The protein could be reconstituted in functional form with efficiency comparable to more traditional methods. Next, the same reaction was utilized to reconstitute MsbA^68^–^70^ – an ABC transporter protein from bacterial cell lysate – into *in situ* formed liposomal membranes. Lastly, NCL reaction was used to accomplish the reconstitution of a mammalian plasma membrane calcium ATPase fusion protein called PMCA2-EGFP^71^ in functional form. This task was particularly challenging because the protein has ten transmembrane domains and can only be expressed in mammalian cells with low yields.^72^ Use of the NCL approach proved to be advantageous over the CuAAC methodology in this particular case because traces of imidazole left over from protein purification inhibited CuAAC but had no significant effect on NCL.

**Fig. 11:**
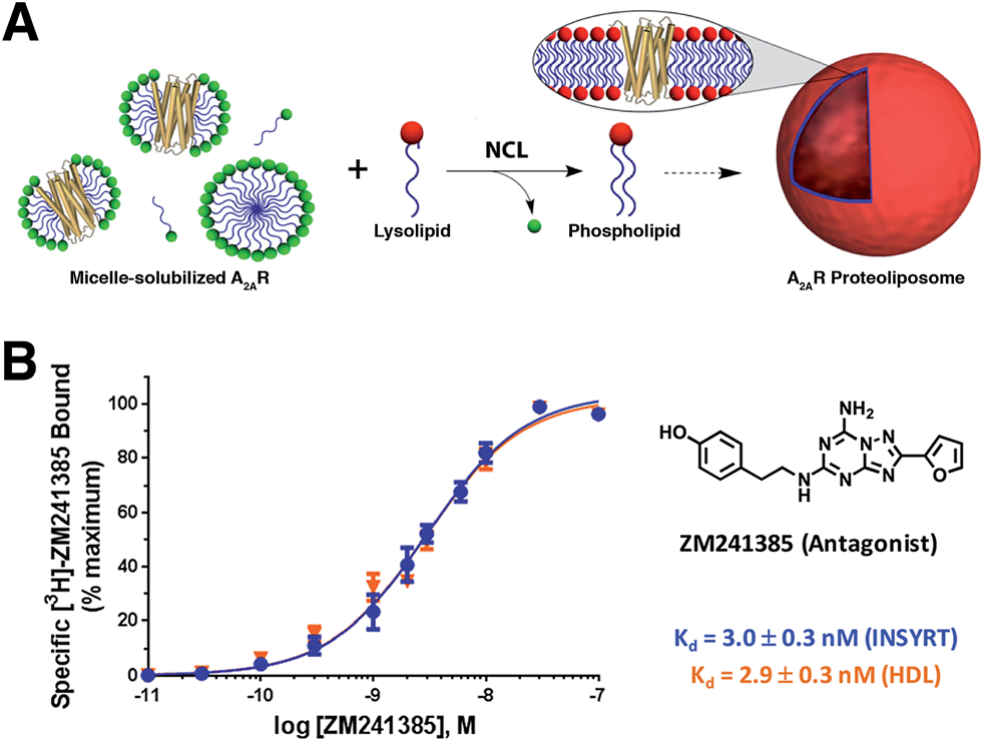
Incorporation of A_2A_R into synthetic liposomes. (A) Schematic representation of the NCL-based phospholipid membrane formation with embedded A_2A_R. (B) [^3^H]-ZM241385 saturation curves with INSYRT reconstituted A_2A_R proteoliposomes (blue line) and A_2A_R–HDL reconstituted nanodiscs (orange line). Radiolabeling experiments showed that both methodologies are similar based on their observed *K*_d_ and *K*_i_.

Brea and co-workers more recently showed that the scope of the NCL-based method could be expanded to incorporate adenosine A_2A_ receptor (A_2A_R) – a G-Protein Coupled Receptor (GPCR)^73^,^74^ – into synthetic membranes formed *in situ* (Fig. 10 and 11).^75^ It is noteworthy that GPCRs constitute the largest class of eukaryotic transmembrane receptor proteins and their study is important for understanding the mechanism of action of many small biomolecules, hormones, and drugs.^76^,^77^ However, current methodologies for their functional reconstitution in biomimetic membranes remains both challenging and limited in scope.^78^ The NCL-based strategy proceeds in the absence of dialysis and/or detergent absorbents, which make it a powerful tool for the assimilation of GPCRs into synthetic liposomes. This methodology, dubbed as INSYRT (*In situ* lipid synthesis for protein reconstitution technology), uses a thioester analog of the commonly used non-ionic detergent *n*-dodecyl-β-d-maltoside (DDM).^79^ Similar to the previous strategies, the DDM thioester plays the dual role of protein solubilizing detergent and phospholipid precursor. The micelle-solubilized A_2A_R is treated with equimolar amount of the cysteine-functionalized lysolipid to form the corresponding phospholipid by NCL, which subsequently leads to stable liposome generation and concurrent embedding of A_2A_R in the membrane. INSYRT proceeds in approximately 20 min without the need for additional post workups or purifications. The reconstituted GPCR was shown to be capable of interacting with known orthosteric ligands, such as the antagonist [^3^H]-ZM241385 (Fig. 11B). Additionally, radiolabeling experiments showed that the reconstitution of A_2A_R during INSYRT yielded similar ligand-binding results when compared to the traditional high-density lipoprotein (HDL)^80^ based reconstitution method as evident from the observed dissociation constant (*K*_d_) and inhibitory constant (*K*_i_) (Fig. 11). The INSYRT methodology could be employed as a powerful general tool for the structural and functional study of membrane proteins.

## 7. Conclusions and future directions

The pursuit of developing synthetic cells from the bottom-up could be rewarding in several ways. First, it may help us answer some of the most fundamental questions pertaining to the very definition of life. For instance, what is the minimum biochemical requirement for life or what were the key steps leading to the origin of life on Earth and possibly elsewhere. In this perspective, we discussed that model systems based on simple coupling reactions between amphiphilic precursors can provide us hints at how complexity arose in early protocell membranes. However, it is still not clear how such activated amphiphiles might have been generated under prebiotic conditions. It has been suggested that ribozymes had once occupied the biochemical niche of fatty acid activating enzymes and acyltransferases;^6^ but no such ribozyme has been discovered so far. So, it stands as an exciting challenge to develop facile routes for fatty acid activation using a macromolecular catalyst (*e.g.* ribozymes or enzymes). Such efforts will help bridging important gaps in the understanding of the evolution of membrane synthesis machinery. Furthermore, such biochemical functionalities can be incorporated into genetic circuits to trigger membrane synthesis and growth in response to a stimulus. In the grand scheme of construction of synthetic life, it is highly desirable to develop a system capable of undergoing Darwinian evolution. It has been shown that simple physical effects can lead to the origin of competition among model protocells.^2^ Also in a separate work, it was shown that spontaneous mutations can be acquired in simple genomic systems over multiple generations in an artificial cell-like compartment.^81^ In the future, it will be highly interesting to combine the above two concepts and develop a vesicular proto-cellular system capable of Darwinian evolution through a combination of acquired genetic mutations and beneficial physical traits. Secondly, artificial cells could serve as model systems for the study of complex biochemical processes. For example, reconstitution of membrane proteins in artificial membranes could be useful for deepening our understanding of how ligands and drugs interact with various receptors in a signaling pathway. Thirdly, we envision that strategies of *de novo* membrane formation can potentially be applied to novel applications in living cells. Lipids remain among the less understood and explored classes of biomolecules. There are multiple disease conditions where aberrations in membrane fluidity, lipid metabolism and signaling pathways are implicated.^82^–^84^ It will be particularly interesting to explore if site-specific or stimuli-responsive lipid synthesis and/or remodeling in cells can ameliorate such conditions. Finally, bottom-up construction of life-like materials will potentially open new avenues for applications in therapeutics, drug delivery, biosensors, and biofuels. Artificial cells could possibly be utilized as smart delivery vehicles for precise spatiotemporal control in targeting and release of the pharmaceutical payloads in disease cells. Moreover, artificial cells could be utilized for rapid and clean synthesis of environmentally friendly fuels. Therefore, the possibilities are endless and will pose exciting challenges to scientists across many disciplines in the future. We believe that various methods of *de novo* vesicle formation will be useful as tools for understanding how membranes can be assembled from basic building blocks and similar ideas could be implemented for studying other self-assembly processes. These strategies serve to demonstrate how synthetic pathways – parallel or even radically different from their biological counterparts – could be incorporated in the approach to bottom-up construction of an artificial cell.

## Conflicts of interest

There are no conflicts to declare.

## References

[cit1] Szostak J. W., Bartel D. P., Luisi P. L. (2001). Nature.

[cit2] Chen I. A., Walde P. (2010). Cold Spring Harbor Perspect. Biol..

[cit3] Whitesides G. M., Boncheva M. (2002). Proc. Natl. Acad. Sci. U. S. A..

[cit4] Monnard P. A., Deamer D. W. (2002). Anat. Rec.

[cit5] Hanczyc M. M., Fujikawa S. M., Szostak J. W. (2003). Science.

[cit6] Blain J. C., Szostak J. W. (2014). Annu. Rev. Biochem..

[cit7] Budin I., Szostak J. W. (2011). Proc. Natl. Acad. Sci. U. S. A..

[cit8] Kraft J. C., Freeling J. P., Wang Z., Ho R. J. (2014). J. Pharm. Sci..

[cit9] Saliba A.-E., Vonkova I., Ceschia S., Findlay G. M., Maeda K., Tischer C., Deghou S., van Noort V., Bork P., Pawson T., Ellenberg J., Gavin A.-C. (2014). Nat. Methods.

[cit10] Noireaux V., Libchaber A. (2004). Proc. Natl. Acad. Sci. U. S. A..

[cit11] Jurak M., Szcześ A., Chibowski E. (2013). Appl. Surf. Sci..

[cit12] Buddingh B. C., van Hest J. C. M. (2017). Acc. Chem. Res..

[cit13] Brea R. J., Hardy M. D., Devaraj N. K. (2015). Chem.–Eur. J..

[cit14] Yao J., Rock C. O. (2013). Biochim. Biophys. Acta.

[cit15] Caschera F., Noireaux V. (2014). Curr. Opin. Chem. Biol..

[cit16] Shindou H., Hishikawa D., Harayama T., Yuki K., Shimizu T. (2009). J. Lipid Res..

[cit17] Deamer D. W., Boatman D. E. (1980). J. Cell Biol..

[cit18] Deamer D. W., Gavino V. (1983). Ann. N. Y. Acad. Sci..

[cit19] Budin I., Debnath A., Szostak J. W. (2012). J. Am. Chem. Soc..

[cit20] Hein J. E., Fokin V. V. (2010). Chem. Soc. Rev..

[cit21] Tornoe C. W., Christensen C., Meldal M. (2002). J. Org. Chem..

[cit22] Rostovtsev V. V., Green L. G., Fokin V. V., Sharpless K. B. (2002). Angew. Chem., Int. Ed..

[cit23] Hardy M. D., Konetski D., Bowman C. N., Devaraj N. K. (2016). Org. Biomol. Chem..

[cit24] Konetski D., Gong T., Bowman C. N. (2016). Langmuir.

[cit25] Zhou C. Y., Wu H., Devaraj N. K. (2015). Chem. Sci..

[cit26] Raibaut L., Ollivier N., Melnyk O. (2012). Chem. Soc. Rev..

[cit27] Dawson P. E., Muir T. W., Clark-Lewis I., Kent S. B. H. (1994). Science.

[cit28] Brea R. J., Cole C. M., Devaraj N. K. (2014). Angew. Chem., Int. Ed..

[cit29] Zhang L., Tam J. P. (1997). Tetrahedron Lett..

[cit30] Tam J. P., Xu J., Eom K. D. (2001). Biopolymers.

[cit31] Brea R. J., Bhattacharya A., Devaraj N. K. (2017). Synlett.

[cit32] Jin Y., Yu C., Denman R. J., Zhang W. (2013). Chem. Soc. Rev..

[cit33] Layer R. W. (1963). Chem. Rev..

[cit34] Minkenberg C. B., Li F., van Rijn P., Florusse L., Boekhoven J., Stuart M. C., Koper G. J., Eelkema R., van Esch J. H. (2011). Angew. Chem., Int. Ed..

[cit35] Takakura K., Yamamoto T., Kurihara K., Toyota T., Ohnuma K., Sugawara T. (2014). Chem. Commun..

[cit36] Wang J., Chen X., Cui W., Yi S. (2015). Colloids Surf., A.

[cit37] Zhu T. F., Szostak J. W. (2009). J. Am. Chem. Soc..

[cit38] Mercier R., Kawai Y., Errington J. (2013). Cell.

[cit39] Takakura K., Toyota T., Sugawara T. (2003). J. Am. Chem. Soc..

[cit40] Kurihara K., Tamura M., Shohda K., Toyota T., Suzuki K., Sugawara T. (2011). Nat. Chem..

[cit41] Kurihara K., Okura Y., Matsuo M., Toyota T., Suzuki K., Sugawara T. (2015). Nat. Commun..

[cit42] Wick R., Walde P., Luisi P. L. (1995). J. Am. Chem. Soc..

[cit43] Hardy M. D., Yang J., Selimkhanov J., Cole C. M., Tsimring L. S., Devaraj N. K. (2015). Proc. Natl. Acad. Sci. U. S. A..

[cit44] Brea R. J., Rudd A. K., Devaraj N. K. (2016). Proc. Natl. Acad. Sci. U. S. A..

[cit45] Varkey J., Isas J. M., Mizuno N., Jensen M. B., Bhatia V. K., Jao C. C., Petrlova J., Voss J. C., Stamou D. G., Steven A. C., Langen R. (2010). J. Biol. Chem..

[cit46] Peter B. J., Kent H. M., Mills I. G., Vallis Y., Butler P. J. G., Evans P. R., McMahon H. T. (2004). Science.

[cit47] Pujals S., Miyamae H., Afonin S., Murayama T., Hirose H., Nakase I., Taniuchi K., Umeda M., Sakamoto K., Ulrich A. S., Futaki S. (2013). ACS Chem. Biol..

[cit48] Stano P., Luisi P. L. (2010). Chem. Commun..

[cit49] Gibellini F., Smith T. K. (2010). IUBMB Life.

[cit50] Schmidli P. K., Schurtenberger P., Luisi P. L. (1991). J. Am. Chem. Soc..

[cit51] Kuruma Y., Stano P., Ueda T., Luisi P. L. (2009). Biochim. Biophys. Acta, Biomembr..

[cit52] Scott A., Noga M. J., de Graaf P., Westerlaken I., Yildirim E., Danelon C. (2016). PLoS One.

[cit53] Murtas G. (2010). Syst. Biol. Synth. Biol..

[cit54] Hartman N. C., Groves J. T. (2011). Curr. Opin. Cell Biol..

[cit55] Linder M. E., Deschenes R. J. (2007). Nat. Rev. Mol. Cell Biol..

[cit56] Paulick M. G., Bertozzi C. R. (2008). Biochemistry.

[cit57] Rudd A. K., Valls Cuevas J. M., Devaraj N. K. (2015). J. Am. Chem. Soc..

[cit58] Peters R. J., Nijemeisland M., van Hest J. C. (2015). Angew. Chem., Int. Ed..

[cit59] Hancock W. O. (2014). Nat. Rev. Mol. Cell Biol..

[cit60] Kholodenko B. N., Hancock J. F., Kolch W. (2010). Nat. Rev. Mol. Cell Biol..

[cit61] Seddon A. M., Curnow P., Booth P. J. (2004). Biochim. Biophys. Acta.

[cit62] Rigaud J.-L., Pitard B., Levy D. (1995). Biochim. Biophys. Acta.

[cit63] Baker M. (2010). Nat. Methods.

[cit64] Cole C. M., Brea R. J., Kim Y. H., Hardy M. D., Yang J., Devaraj N. K. (2015). Angew. Chem., Int. Ed..

[cit65] Yoshikawa S., Muramoto K., Shinzawa-Itoh K., Mochizuki M. (2012). Biochim. Biophys. Acta.

[cit66] Vik S. B., Capaldi R. A. (1977). Biochemistry.

[cit67] Yoshikawa S., Muramoto K., Shinzawa-Itoh K. (2011). Biochim. Biophys. Acta, Bioenerg..

[cit68] Doshi R., Ali A., Shi W., Freeman E. V., Fagg L. A., van Veen H. W. (2013). J. Biol. Chem..

[cit69] Eckford P. D., Sharom F. J. (2008). J. Biol. Chem..

[cit70] Haubertin D. Y., Madaoui H., Sanson A., Guerois R., Orlowski S. (2006). Biophys. J..

[cit71] Chicka M. C., Strehler E. E. (2003). J. Biol. Chem..

[cit72] Strehler E. E., Zacharias D. A. (2001). Physiol. Rev..

[cit73] Katritch V., Cherezov V., Stevens R. C. (2013). Annu. Rev. Pharmacol. Toxicol..

[cit74] Salon J. A., Lodowski D. T., Palczewski K. (2011). Pharmacol. Rev..

[cit75] Brea R. J., Cole C. M., Lyda B. R., Ye L., Prosser R. S., Sunahara R. K., Devaraj N. K. (2017). J. Am. Chem. Soc..

[cit76] Pierce K. L., Premont R. T., Lefkowitz R. J. (2002). Nat. Rev. Mol. Cell Biol..

[cit77] Kobilka B. K. (2007). Biochim. Biophys. Acta.

[cit78] Grisshammer R. (2009). Methods Enzymol..

[cit79] Rosevear P., VanAken T., Baxter J., Ferguson-Miller S. (1980). Biochemistry.

[cit80] Bocquet N., Kohler J., Hug M. N., Kusznir E. A., Rufer A. C., Dawson R. J., Hennig M., Ruf A., Huber W., Huber S. (2015). Biochim. Biophys. Acta, Biomembr..

[cit81] Ichihashi N., Usui K., Kazuta Y., Sunami T., Matsuura T., Yomo T. (2013). Nat. Commun..

[cit82] Wymann M. P., Schneiter R. (2008). Nat. Rev. Mol. Cell Biol..

[cit83] Vellodi A. (2005). Br. J. Haematol..

[cit84] Maxfield F. R., Tabas I. (2005). Nature.

